# Detection of *Mycobacterium tuberculosis* complex DNA in CD34-positive peripheral blood mononuclear cells of asymptomatic tuberculosis contacts: an observational study

**DOI:** 10.1016/S2666-5247(21)00043-4

**Published:** 2021-06

**Authors:** Mulugeta Belay, Begna Tulu, Sidra Younis, David A Jolliffe, Dawit Tayachew, Hana Manwandu, Tenagnework Abozen, Emawayish A Tirfie, Metasebia Tegegn, Aboma Zewude, Sally Forrest, Jonathan Mayito, Jim F Huggett, Gerwyn M Jones, Denise M O'Sullivan, Henny M Martineau, Mahdad Noursadeghi, Aneesh Chandran, Kathryn A Harris, Vlad Nikolayevskyy, Julie Demaret, Stefan Berg, Martin Vordermeier, Taye T Balcha, Abraham Aseffa, Gobena Ameni, Markos Abebe, Stephen T Reece, Adrian R Martineau

**Affiliations:** a Barts and the London School of Medicine and Dentistry, Queen Mary University of London, London, UK; b Armauer Hansen Research Institute, Addis Ababa, Ethiopia; c Institute of Health and Society, University of Oslo, Oslo, Norway; d Aklilu Lemma Institute of Pathobiology, Addis Ababa University, Addis Ababa, Ethiopia; e Department of Medical Laboratory Science, Bahir Dar University, Bahir Dar, Ethiopia; f National University of Medical Sciences, Rawalpindi, Punjab, Pakistan; g Department of Medicine, University of Cambridge, Cambridge, UK; h School of Biomedical Sciences, Makerere University College of Health Sciences, Kampala, Uganda; i National Measurement Laboratory, LGC, Teddington, Middlesex, UK; j School of Biosciences & Medicine, Faculty of Health & Medical Science, University of Surrey, Guildford, UK; k Department of Pathology, The Royal Veterinary College, Hatfield, UK; l Division of Infection and Immunity, University College London, London, UK; m Camelia Botnar Laboratories, Great Ormond Street Hospital for Children, London, UK; n National Mycobacterium Reference Service—South, National Infection Service, London, UK; o Institut d'Immunologie, Centre de Biologie-Pathologie-Génétique du CHRU de Lille, Lille, France; p Animal and Plant Health Agency, New Haw, UK; q Clinical Infection Medicine, Department of Translational Medicine, Lund University, Malmö, Sweden; r Department of Veterinary Medicine, College of Food and Agriculture, United Arab Emirates University, Al Ain, United Arab Emirates; s Kymab, Babraham Research Campus, Cambridge, UK

## Abstract

**Background:**

Haematopoietic stem cells expressing the CD34 surface marker have been posited as a niche for *Mycobacterium tuberculosis* complex bacilli during latent tuberculosis infection. Our aim was to determine whether *M tuberculosis* complex DNA is detectable in CD34-positive peripheral blood mononuclear cells (PBMCs) isolated from asymptomatic adults living in a setting with a high tuberculosis burden.

**Methods:**

We did a cross-sectional study in Ethiopia between Nov 22, 2017, and Jan 10, 2019. Digital PCR (dPCR) was used to determine whether *M tuberculosis* complex DNA was detectable in PBMCs isolated from 100 mL blood taken from asymptomatic adults with HIV infection or a history of recent household or occupational exposure to an index case of human or bovine tuberculosis. Participants were recruited from HIV clinics, tuberculosis clinics, and cattle farms in and around Addis Ababa. A nested prospective study was done in a subset of HIV-infected individuals to evaluate whether administration of isoniazid preventive therapy was effective in clearing *M tuberculosis* complex DNA from PBMCs. Follow-up was done between July 20, 2018, and Feb 13, 2019. QuantiFERON-TB Gold assays were also done on all baseline and follow-up samples.

**Findings:**

Valid dPCR data (ie, droplet counts >10 000 per well) were available for paired CD34-positive and CD34-negative PBMC fractions from 197 (70%) of 284 participants who contributed data to cross-sectional analyses. *M tuberculosis* complex DNA was detected in PBMCs of 156 of 197 participants with valid dPCR data (79%, 95% CI 74–85). It was more commonly present in CD34-positive than in CD34-negative fractions (154 [73%] of 197 *vs* 46 [23%] of 197; p<0·0001). Prevalence of dPCR-detected *M tuberculosis* complex DNA did not differ between QuantiFERON-negative and QuantiFERON-positive participants (77 [78%] of 99 *vs* 79 [81%] of 98; p=0·73), but it was higher in HIV-infected than in HIV-uninfected participants (67 [89%] of 75 *vs* 89 [73%] of 122, p=0·0065). By contrast, the proportion of QuantiFERON-positive participants was lower in HIV-infected than in HIV-uninfected participants (25 [33%] of 75 *vs* 73 [60%] of 122; p<0·0001). Administration of isoniazid preventive therapy reduced the prevalence of dPCR-detected *M tuberculosis* complex DNA from 41 (95%) of 43 HIV-infected individuals at baseline to 23 (53%) of 43 after treatment (p<0·0001), but it did not affect the prevalence of QuantiFERON positivity (17 [40%] of 43 at baseline *vs* 13 [30%] of 43 after treatment; p=0·13).

**Interpretation:**

We report a novel molecular microbiological biomarker of latent tuberculosis infection with properties that are distinct from those of a commercial interferon-γ release assay. Our findings implicate the bone marrow as a niche for *M tuberculosis* in latently infected individuals. Detection of *M tuberculosis* complex DNA in PBMCs has potential applications in the diagnosis of latent tuberculosis infection, in monitoring response to preventive therapy, and as an outcome measure in clinical trials of interventions to prevent or treat latent tuberculosis infection.

**Funding:**

UK Medical Research Council.

## Introduction

The *Mycobacterium tuberculosis* complex is a genetically homogeneous group of organisms whose members include *M tuberculosis* and *Mycobacterium bovis.* They cause active tuberculosis in humans, cattle, and other animals, which imposes a major toll on health and economic growth that is borne disproportionately by low-income countries.^1^ Tuberculosis commonly arises following reactivation of latent infection. The global prevalence of latent tuberculosis infection is not known, because there is no gold standard microbiological test to detect it. Diagnosis of latent tuberculosis infection is predicated on the detection of cell-mediated immune responses to *M tuberculosis* complex, which can be elicited in vivo (by intradermal injection of *M tuberculosis* complex antigens in tuberculin skin tests) or ex vivo (by stimulation of peripheral blood leucocytes with *M tuberculosis* complex-specific antigens in interferon-γ [IFN-γ] release assays [IGRAs]). On the basis of the results of these tests, global prevalence of latent tuberculosis infection has been estimated at 23%,^2^ with prevalence in high burden settings, such as Ethiopia, being nearly three times higher.^3^

**Research in context**

**Evidence before this study**

We searched PubMed for research articles published in English from database inception to Nov 21, 2017, using the terms “latent tuberculosis”, “haematopoietic stem cell”, “CD34”, “PCR”, and “human”. We found one report of *Mycobacterium tuberculosis* DNA being detected in CD34-positive peripheral blood mononuclear cells (PBMCs) of 15 HIV-uninfected adults with latent tuberculosis living in Austria. We did not find reports of this observation being investigated in a high tuberculosis burden setting, or in HIV-infected people.

**Added value of this study**

We report that *M tuberculosis* complex-derived genomic DNA can be readily detected in PBMCs of asymptomatic adults living in Ethiopia, by applying a highly sensitive digital PCR (dPCR) assay to material isolated from a large (100 mL) blood draw. Where present, *M tuberculosis* complex DNA was found more frequently in CD34-positive versus CD34-negative PBMCs, implicating haematopoietic stem cells as a niche for *M tuberculosis* during latent tuberculosis infection. Prevalence of dPCR-detected *M tuberculosis* complex DNA did not differ between interferon-γ release assays (IGRA)-negative and IGRA-positive participants, but it was higher in HIV-infected than in HIV-uninfected participants. By contrast, prevalence of IGRA-positivity was lower in HIV-infected than in HIV-uninfected participants. In a nested prospective study, isoniazid preventive therapy rendered *M tuberculosis* complex DNA undetectable in 44% of individuals in whom it was detected at baseline; no statistically significant change in the proportion of IGRA-positive individuals was seen following administration of isoniazid preventive therapy.

**Implications of all the available evidence**

We report a novel molecular microbiological biomarker of latent tuberculosis infection with properties that are distinct from those of an IGRA. Detection of *M tuberculosis* complex DNA in PBMCs has potential applications in the diagnosis of latent tuberculosis infection, in monitoring response to preventive therapy and as an outcome measure in clinical trials of interventions to prevent or treat latent tuberculosis infection. Longitudinal studies to evaluate the prognostic significance of dPCR-detected *M tuberculosis* complex DNA in PBMCs of asymptomatic adults are needed.

Effective antimicrobial treatments for latent tuberculosis infection (preventive therapy) are available, and their widespread roll-out will be needed if WHO's goal of tuberculosis elimination by 2050 is to be achieved.^4^ However, limitations of existing IGRAs represent a barrier to effective targeting of preventive therapy: their sensitivities and specificities are imperfect, they cannot determine whether an individual is infected with a drug-sensitive or a drug-resistant isolate, and they do not revert to negative after treatment.^5^, ^6^ Development of a microbiological test for latent tuberculosis infection with greater sensitivity and specificity, which could be used to detect drug resistance and evaluate response to treatment would allow preventive therapy to be better targeted and more effective. It would also be a valuable research tool with potential to provide insights into tuberculosis pathogenesis and to be used as an outcome measure for trials of interventions seeking to prevent or treat latent tuberculosis infection.

Historically, microbiological evidence of latent tuberculosis infection could only be detected using cadaveric tissue.^7^ However, studies have reported that haematopoietic stem cells, which bear the CD34 glycoprotein as a surface marker, might represent a niche for *M tuberculosis* complex during latent tuberculosis infection.^8^, ^9^ Therefore, *M tuberculosis* complex could be detected in peripheral blood, because hematopoietic stem cells transition between the bone marrow and the circulation.^10^ In keeping with this hypothesis, two small clinical studies have reported detection of *M tuberculosis* complex DNA in the peripheral blood of 11 IGRA-positive but asymptomatic HIV-uninfected adults living in northern Europe using PCR.^8^, ^11^ To date, this finding has not been evaluated in settings with a high tuberculosis burden or characterised at scale. We therefore aimed to determine whether *M tuberculosis* complex DNA could be detected in CD34-positive and CD34-negative peripheral blood mononuclear cells (PBMCs) isolated from a heterogeneous group of asymptomatic adults in whom active tuberculosis had been excluded.

## Methods

### Study design and participants

Asymptomatic study participants were recruited to a cross-sectional study at health centres and hospitals in Addis Ababa and from commercial cattle farms in its vicinity. Household tuberculosis contacts were identified by contact tracing of index cases of smear-positive pulmonary tuberculosis presenting to one of 21 outpatient clinics in Addis Ababa. Participants with exposure to cattle with bovine tuberculosis were identified at 16 farms in and around Addis Ababa. HIV-infected participants were recruited at a single outpatient clinic at the ALERT hospital, Addis Ababa. Adults screened at health centres and hospitals were eligible to take part if they had been in regular household contact with an index case of smear-positive pulmonary tuberculosis over the previous month or if they were known to have HIV infection (irrespective of contact history). Individuals identified at cattle farms were eligible to take part if they had been exposed to a cow exhibiting a strong reaction to a comparative intradermal tuberculin test (performed as previously described)^12^ and found to have culture-positive bovine tuberculosis at autopsy. Exclusion criteria were age less than 18 years, symptoms of active tuberculosis, current prescription of anti-tuberculosis medication, and inability to travel to a participating x-ray facility for exclusion of active tuberculosis. Women of childbearing potential had a urine pregnancy test (Medi-Test; BHR Pharmaceuticals; Nuneaton, UK) and were excluded if it was positive. All potential participants were screened for anaemia using a point-of-care test (HemoCue Hemoglobin 301 Analyser; HemoCue; Ängelholm, Sweden) on a finger prick blood sample; those with a haemoglobin concentration of less than 10 g/dL were excluded. A chest radiograph was also done, and participants with any radiographic abnormality consistent with active tuberculosis were excluded and referred to the National Tuberculosis Treatment Program for further clinical assessment and treatment if necessary.

In addition to this cross-sectional study, we did a nested prospective study in HIV-infected participants who did not have any contraindication to isoniazid preventive therapy. This group was offered a 6-month course of 300 mg isoniazid (Cadila Pharmaceuticals; Ahmedabad, India) and 50 mg pyridoxine (Just Vitamins; Coventry, UK) daily and invited to return to give a second 104 mL blood sample for the same laboratory tests following treatment completion.

The study was approved by the Ethiopian National Research Ethics Review Committee, Addis Ababa, Ethiopia (310/253/2017) and by the Queen Mary Ethics of Research Committee, London, UK (QMERC 2017/07). Written informed consent was obtained from all participants.

### Procedures

Participants completed a questionnaire detailing age, sex, past medical history, medication use, previous exposure to tuberculosis, BCG vaccination status, and smoking history. A blood sample was drawn into sodium heparin tubes (BD; Franklin Lakes, NJ, USA; 100 mL) and QuantiFERON-TB-Gold Plus blood collection tubes (Qiagen; Hilden, Germany; 4 × 1 mL) for laboratory tests.

Full details of laboratory methods are provided in the appendix (pp 2–7). To minimise risk of cross-contamination, all laboratory work was done in biosafety level 2 laboratories that had never been used for work with *M tuberculosis* complex organisms. QuantiFERON-TB Gold Plus assays (Qiagen) were done according to the manufacturer's instructions. PBMCs were isolated from 100 mL blood over HistoPaque-1077 (Sigma-Aldrich; St Louis, MO, USA). CD34-positive and CD34-negative PBMCs were separated using CD34 MicroBead Ultra-Pure Kits and MS columns (both from Miltenyi Biotec; Bergisch Gladbach, Germany) according to the manufacturer's instructions. Effective enrichment or depletion of CD34-positive PBMCs from positively or negatively selected cell fractions was confirmed by flow cytometry in one donor (appendix p 14).

To investigate whether cross-contamination might have occurred during PBMC processing in the biosafety level 2 laboratory in Addis Ababa, five healthy personnel from the study team travelled to Ethiopia and gave blood samples for isolation of PBMCs and separation of CD34-positive and CD34-negative fractions in this laboratory. The same five donors returned to the UK to give a second blood sample 1–2 months later, for isolation of PBMCs and separation of CD34-positive and CD34-negative fractions in the biosafety level 2 laboratory in London.

DNA was extracted from CD34-positive and CD34-negative cell pellets using a cetyltrimethylammonium bromide and chloroform-isoamyl alcohol protocol,^13^ and concentrations of DNA in cell extracts were determined using the NanoDrop 8000 spectrophotometer (ThermoFisher Scientific; Waltham, MA, USA). Effectiveness of this protocol for extraction of DNA from intracellular *M tuberculosis* was confirmed (appendix p 15). To ensure equal sensitivity for detection of *M tuberculosis* DNA in CD34-negative and CD34-positive fractions, the extract from the CD34-negative cell pellet from each participant was diluted so that its DNA concentration matched that of the corresponding extract from the CD34-positive cell pellet. Thus, *M tuberculosis* DNA copy number was compared for equivalent amounts of human DNA, which will have approximated to equivalent numbers of host cells (because *M tuberculosis* DNA will have made up a negligible proportion of total DNA). Digital PCRs (dPCRs) were prepared using 10 μL of extract, and analysed using the QX200 AutoDG Droplet Digital PCR System (Bio-Rad Laboratories; Hercules, CA, USA) to detect DNA of two *M tuberculosis* complex-specific DNA sequences: the multi-copy insertion sequence (IS) *6110* and the single-copy gene *rpoB*.^14^ dPCR offers a better signal-to-noise ratio than quantitative PCR (qPCR) when target DNA is at a low concentration relative to non-target DNA.^15^ dPCR data from wells with droplet counts of less than 10 000 were disregarded, as per the manufacturer's instructions. dPCR data are presented as copy number per 20 μL. Oligonucleotide information, thermal cycling conditions, and a Digital MIQE (Minimum Information for Publication of Quantitative Digital PCR Experiments) checklist^16^ are provided in the appendix (appendix pp 8–12). Results of a dilution series experiment to detect 2–2000 copies of genomic *M tuberculosis* DNA using dPCR assays for IS*6110* and *rpoB* are presented in the appendix (appendix p 16).

We did several control experiments and analyses to investigate non-specific amplification and cross-contamination with *M tuberculosis* DNA or amplicons as causes of false-positive results as described in the appendix (p 6).

### Statistical analysis

Sample size was based on the power required to estimate prevalence of *M tuberculosis* complex DNA carriage for participants recruited at tuberculosis clinics, cattle farms, and HIV clinics. Estimating prevalence at 60%,^3^ we calculated that 93 participants in each group would need to be studied to estimate prevalence at the 95% confidence level with a desired precision of 0·10. Sample size for the nested prospective study in participants receiving isoniazid preventive therapy was based on the number of participants needed to show a reduction in the mean number of *M tuberculosis* complex DNA element copies detectable in CD34-positive cells isolated from 100 mL of blood before and after isoniazid treatment. Assuming that the mean number of copies of *M tuberculosis* complex at baseline would be 4·3 in dPCR-positive participants, with standard deviation 2·5,^8^ we calculate that a total of 43 dPCR-positive participants would need to be followed to detect a 25% reduction in mean copy number per 20 μL well (from 4·3 to 3·2) with 80% power at the 5% significance level.

Statistical analyses were done using Stata/IC version 12.1. Outcomes were the proportion of individuals in whom *M tuberculosis* complex DNA was detectable (categorical variable), and the number of copies of IS*6110* and *rpoB* per 20 μl well (continuous variable). Estimates of prevalence of detectable *M tuberculosis* complex DNA are presented with 95% CIs. The limits of detection for IS*6110* and *rpoB* were calculated using the formula:^17^

$$\text{Limit of detection}=\text{limit of blank}+1ċ645\times {\text{SD}}_{\text{low concentration sample}}$$

where:

$$\text{Limit of blank}={\text{mean}}_{\text{blank}}+1ċ645\times {\text{SD}}_{\text{blank}}$$

This calculation yielded values of 4·38 copies per 20 μL well for IS*6110* and 3·80 copies per 20 μL well for *rpoB*; values below these thresholds were assigned a zero value in statistical analyses. Individuals for whom valid dPCR data were unavailable for paired CD34-positive and CD34-negative PBMCs were excluded from statistical analyses. dPCRs were considered positive if DNA of IS*6110* or *rpoB* was detectable. Categorical QuantiFERON status was assigned on the basis of IFN-γ concentrations in supernatants in antigen-stimulated tubes and positive and negative controls using QuantiFERON-TB Gold Plus Analysis Software (Qiagen). IFN-γ concentrations in supernatants of antigen-stimulated whole blood were also analysed as continuous variables when testing for correlations with IS*6110* and *rpoB* copy numbers. Fisher's exact tests and McNemar's tests were used to analyse contingency tables for unpaired and paired categorical data, respectively. Mann-Whitney U tests and Wilcoxon matched-pairs signed rank tests were used for unpaired and paired comparisons of continuous variables, respectively. Paired analyses were done to compare data relating to the same individual (eg, comparison of *M tuberculosis* complex DNA copy numbers in different cell fractions from the same individual or comparison of *M tuberculosis* complex DNA copy numbers in the same individual sampled at baseline and follow-up). Unpaired analyses were done to compare data from different individuals (eg, comparison of *M tuberculosis* complex DNA copy numbers in QuantiFERON-negative and QuantiFERON-positive individuals). Spearman's rank correlation coefficient was used to evaluate correlation between copy numbers per 20 μL well for IS*6110* compared with *rpoB*. Statistical significance was inferred where p values were less than 0·05.

### Role of the funding source

The funder of the study had no role in study design, data collection, data analysis, data interpretation, or writing of the report.

## Results

284 participants were enrolled between Nov 22, 2017, and Jan 10, 2019. Follow-up visits occurred between July 20, 2018, and Feb 13, 2019. Valid dPCR data (ie, droplet counts >10 000 per well) were available for paired CD34-positive and CD34-negative PBMCs from 197 (69%) of them. Characteristics of these 197 participants, who contributed data to cross-sectional analyses, are presented in the table. All 75 HIV-infected participants were offered isoniazid preventive therapy, of whom 56 had valid dPCR data at baseline, took isoniazid preventive therapy, and attended 6-month follow-up. Valid dPCR data from 6 months after isoniazid preventive therapy were available for 43 (77%) of 56.

**Table:** Participant characteristics

		**Study participants (N=197)**
Age, years		34 (24–40)
Sex		
	Female	95 (48%)
	Male	102 (52%)
Study group		
	Recent household exposure to human index case with smear-positive pulmonary tuberculosis, Addis Ababa	69 (35%)*
	Recent occupational exposure to bovine index case with culture-positive bovine tuberculosis, recruited in cattle farms	56 (28%)†
	Attending outpatient HIV clinic, Addis Ababa	72 (37%)‡
BCG scar		78 (40%)
Current smoker		8 (4%)
HIV infection		75 (38%)§
QuantiFERON-positive		98 (50%)¶

Data are median (IQR) or n (%).

* Three (4%) of 69 had HIV infections.

† 0 (0%) of 56 had HIV infections, and 10 (18%) had recent household tuberculosis contact.

‡ 72 (100%) of 72 had HIV infections, five (7%) had recent household tuberculosis contact, and 14 (19%) had previously had active tuberculosis.

§ Viral load was undetectable in 59 (92%) of 64 HIV-infected individuals for whom data were available.

¶ 25 (26%) of 98 had HIV infections, and 40 (41%) had a BCG scar.

**Figure 1:**
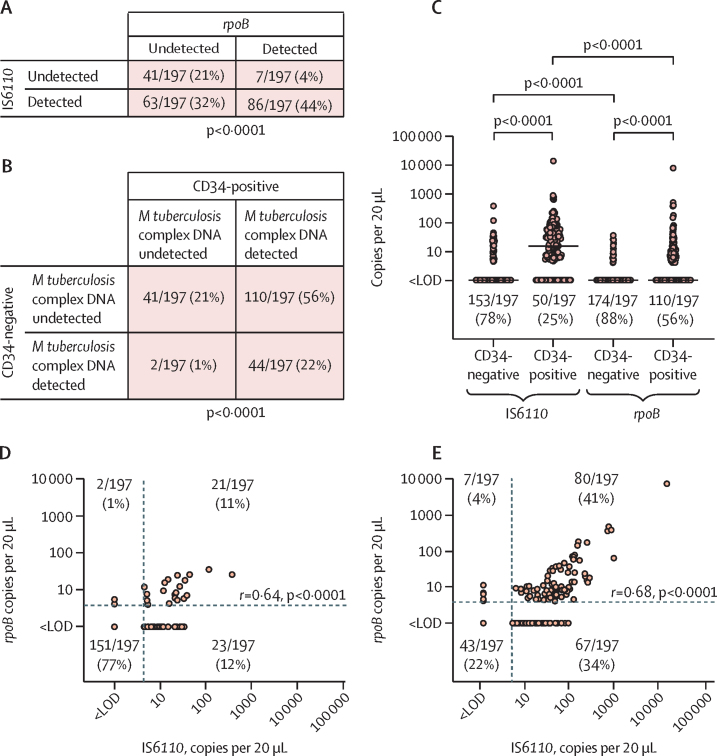
Detection of *Mycobacterium tuberculosis* complex DNA in PBMCs of asymptomatic adults Detection of *rpoB* by detection of IS*6110* (A), and detection of *M tuberculosis* complex DNA in CD34-positive versus CD34-negative PBMCs (B); p values were calculated with Fisher's exact test. (C) IS*6110* and *rpoB* copy numbers per 20 μL well detected in CD34-negative versus CD34-positive PBMCs; p values were calculated with Wilcoxon matched-pairs signed rank tests. Lines show median values and proportions with undetectable *M tuberculosis* complex DNA are displayed below the scatter plots for each condition. IS*6110* versus *rpoB* copy number per 20 μL well for (D) CD34-negative PBMCs and (E) CD34-positive PBMCs; correlation coefficients and p values were calculated with Spearman's tests. Proportions of participants occupying each quadrant are also displayed. LOD=limit of detection. PBMC=peripheral blood mononuclear cell.

DNA for IS*6110* or *rpoB* was detected in CD34-positive or CD34-negative PBMC fractions from 156 of 197 participants (79·2%; 95% CI 73·5–84·9; figure 1A). Proportions of participants in whom IS*6110* and *rpoB* were detected in CD34-positive or CD34-negative PBMC fractions are presented separately in the appendix (p 13). Representative amplification plots are also shown (appendix pp 17–19). *M tuberculosis* complex DNA was detected more commonly in CD34-positive than in CD34-negative fractions (154 [73%] of 197 *vs* 46 [23%] of 197; p<0·0001; figure 1B). When data for IS*6110* and *rpoB* were analysed separately, IS*6110* was detected more frequently than *rpoB* in both CD34-negative PBMCs (44 [22%] of 197 *vs* 23 [12%] of 197; p<0·0001) and CD34-positive PBMCs (147 [75%] of 197 *vs* 87 [44%] of 197; p<0·0001). The copy number per 20 μL well for IS*6110* and *rpoB* was larger in CD34-positive than in CD34-negative fractions, and IS*6110* copy number per 20 μL well was larger than *rpoB* copy number per 20 μL well in both CD34-negative and CD34-positive PBMCs (p<0·0001 for all comparisons; figure 1C). Copy numbers per 20 μL well for IS*6110* and *rpoB* were positively correlated in both CD34-negative and CD34-positive fractions (p<0·0001 for both correlations; figure 1D, E). The proportion of participants in whom *M tuberculosis* complex DNA was detectable did not differ between those with and without a BCG scar (62 [79%] of 78 *vs* 94 [79%] of 119; p>0·99).

In control experiments investigating cross-contamination with *M tuberculosis* DNA, amplicons, or non-specific amplification as causes of false-positive results, ten clinical samples that were positive for both *rpoB* and IS*6110* with our dPCR assay were also positive for *ESAT6* as detected using qPCR in an independent laboratory (cycle threshold ≤32; appendix p 20). Moreover, cycle threshold for *ESAT6* correlated negatively with *rpoB* copy number from dPCR (p=0·0022; appendix p 20). dPCR analysis of triplicate swabs of benches and microbiological safety cabinets in the Ethiopian laboratory where blood samples were processed revealed that IS*6110* and *rpoB* were undetectable in nine (100%) of nine swabs analysed. Additionally, analysis of paired blood samples from five healthy donors that were processed in Addis Ababa and London revealed that IS*6110* and *rpoB* were undetectable in all PBMC samples isolated in Ethiopia, and in all but one PBMC sample isolated in the UK (one of three triplicates for one donor was positive at 5·9 copies per 20 μL well). Plots of IS*6110* and *rpoB* copy numbers showed that PCR-positive samples were distributed evenly over the course of the study (appendix p 20). Both IS*6110* and *rpoB* were undetectable in 32 of 32 replicates of a reference human DNA sample. dPCR assays for IS*6110* and *rpoB* using DNA from non-tuberculous mycobacteria (*Mycobacterium abscessus* and *Mycobacterium szulgai*) and some common skin commensals (*Staphylococcus epidermidis, Staphylococcus aureus*, and *Streptococcus pyogenes*) were negative in all instances. dPCR assays for targets in *S pyogenes* (*csrR*), *S aureus* (*coA*), and *S epidermidis* (*femA_SE*) were negative for 20 of 20 extracts tested. DNA for IS*6110* and *rpoB* was undetectable in 10 of 10 PBMC samples that had been spiked with UltraPure MicroBeads (Miltenyi Biotech; Cologne, Germany).

**Figure 2:**
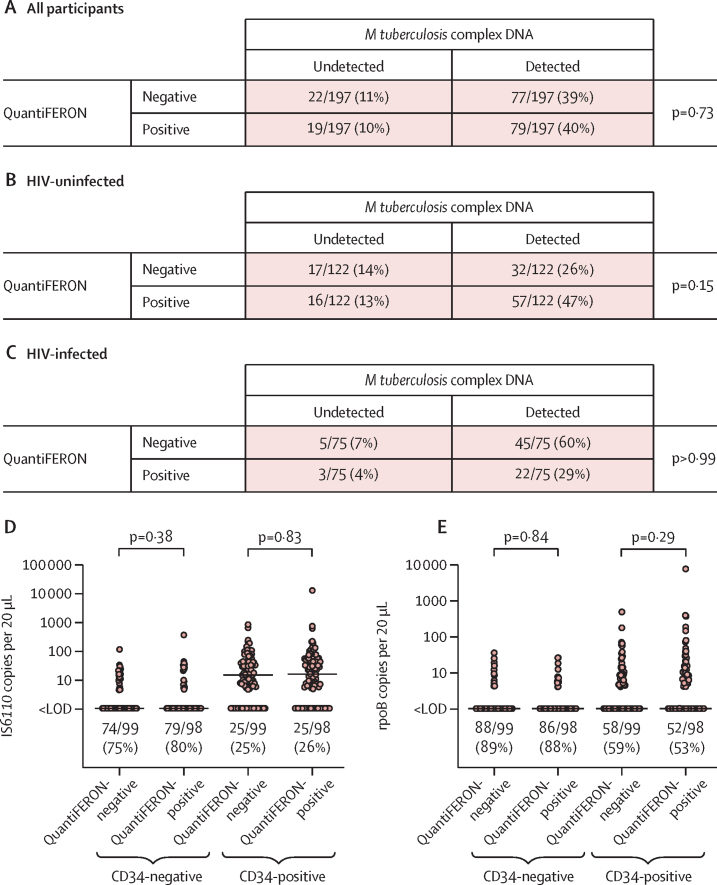
Detection of *Mycobacterium tuberculosis* complex DNA in PBMCs of asymptomatic adults by QuantiFERON status Detection of *M tuberculosis* complex DNA by QuantiFERON status in all participants (A), HIV-uninfected participants (B), and HIV-infected participants (C). (D) IS*6110* copy number per 20 μL well in CD34-negative and CD34-positive PBMCs. (E) *rpoB* copy number per 20 μL well in CD34-negative and CD34-positive PBMCs. p values for contingency tables were calculated with Fisher's exact tests. p values for comparison of continuous variables were calculated with Mann-Whitney tests. In panels D and E, proportions with undetectable *M tuberculosis* complex DNA are displayed below the scatter plots for each condition, and lines show median values. LOD=limit of detection. PBMC=peripheral blood mononuclear cell.

Next, we investigated whether detection of *M tuberculosis* complex DNA in PBMC was associated with QuantiFERON status. The proportion of participants in whom *M tuberculosis* complex DNA was detectable did not differ between QuantiFERON-negative and QuantiFERON-positive individuals in the study population as a whole (77 [78%] of 99 *vs* 79 [81%] of 98; p=0·73; figure 2A), or when the analysis was stratified by HIV status (figure 2B, C). Moreover, copy numbers of IS*6110* and *rpoB* per 20 μL well did not differ by QuantiFERON status in either CD34-negative or CD34-positive PBMCs (p≥0·29; figure 2D, E). No correlation was seen between antigen-stimulated supernatant concentrations of IFN-γ and IS*6110* or *rpoB* copy numbers per 20 μL well (appendix p 21).

**Figure 3:**
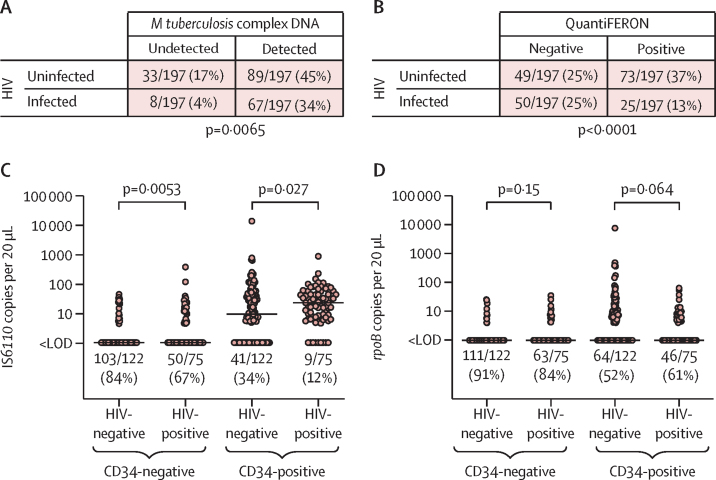
Detection of *Mycobacterium tuberculosis* complex DNA in PBMCs of asymptomatic adults by HIV status Detection of *M tuberculosis* complex DNA by HIV status (A), and QuantiFERON status by HIV status (B); p values were calculated with Fisher's exact test. IS*6110* (C) and *rpoB* (D) copy numbers per 20 μL well in CD34-negative and CD34-positive PBMCs; p values were calculated with Mann-Whitney tests. In panels C and D, lines show median values and proportions with undetectable *M tuberculosis* complex DNA are displayed below the scatter plots for each condition. LOD=limit of detection. PBMC=peripheral blood mononuclear cell.

We then proceeded to test for an association between presence of *M tuberculosis* complex DNA and infection with HIV. IS*6110* or *rpoB* was detected in PBMCs from 67 (89%) of 75 HIV-infected participants and in 89 (73%) of 122 HIV-uninfected participants (p=0·0065; figure 3A). By contrast, QuantiFERON-positivity was less common in HIV-infected than in HIV-uninfected participants (25 [33%] of 75 *vs* 73 [60%] of 122; p<0·0001; figure 3B). When the cross-tabulation of PCR status by QuantiFERON status was stratified according to HIV status, we observed that 45 (58%) of 77 QuantiFERON-negative, PCR-positive individuals had HIV infections, as compared with three (16%) of 19 QuantiFERON-positive, PCR-negative individuals (p<0·0001; figure 2A, C). IS*6110* copy number per 20 μL well was higher in both CD34-positive and CD34-negative PBMCs isolated from HIV-infected participants than in cells isolated from HIV-uninfected participants (p≤0·027; figure 3C). Statistically significant differences in *rpoB* copy number per 20 μL well in HIV-infected compared with HIV-uninfected individuals were not seen (p≥0·064; figure 3D). Among HIV-infected participants, no differences in copy numbers of IS*6110* or *rpoB* per 20 μL well were seen according to HIV viral load or previous history of active tuberculosis (appendix pp 22–23).

**Figure 4:**
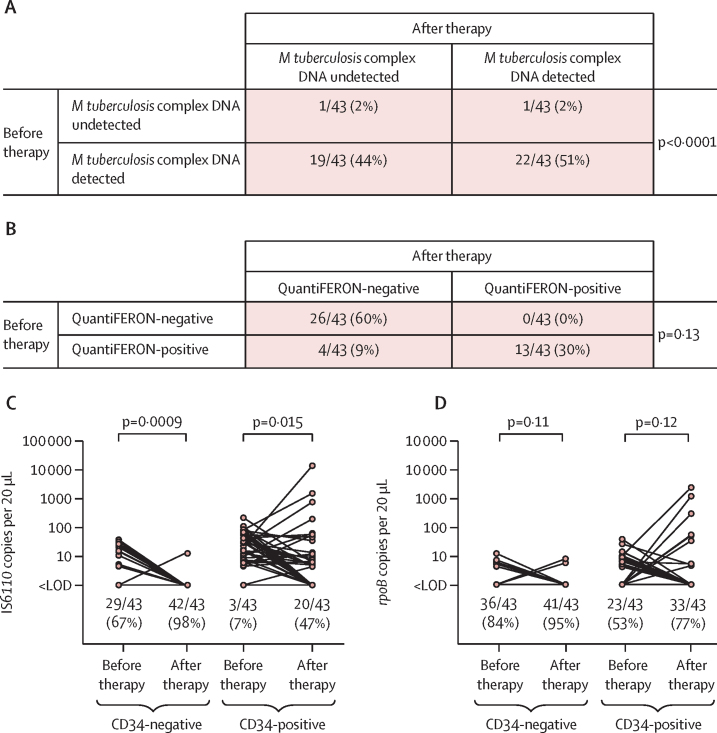
Detection of *Mycobacterium tuberculosis* complex DNA in HIV-infected adults before and after isoniazid preventive therapy Detection of *M tuberculosis* complex DNA (A) and QuantiFERON status (B) after therapy versus before therapy; p value were calculated with McNemar's test. IS*6110* (C) and *rpoB* (D) copy numbers per 20 μL well in CD34-negative and CD34-positive PBMCs; p values were calculated with Wilcoxon matched-pairs signed rank tests. In panels C and D, lines join datapoints for individual participants, and proportions with undetectable *M tuberculosis* complex DNA are displayed below the scatter plots for each condition. LOD=limit of detection.

Analysis of data from our nested prospective study showed that isoniazid preventive therapy reduced the proportion of HIV-infected individuals in whom *M tuberculosis* complex DNA was detectable from 41 (95%) of 43 at baseline to 23 (53%) of 43 after treatment (p<0·0001; figure 4A). By contrast, the proportion of participants who were QuantiFERON-positive did not significantly differ (17 [40%] of 43 at baseline *vs* 13 [30%] of 43 after treatment; p=0·13; figure 4B). Isoniazid preventive therapy reduced IS*6110* copy number per 20 μL well in both CD34-negative and CD34-positive PBMCs (p≤0·015; figure 4C), but statistically significant differences in *rpoB* copy number per 20 μL well before and after isoniazid preventive therapy were not seen (p≥0·11; figure 4D).

## Discussion

We report findings of the first study to test for the presence of *M tuberculosis* complex DNA in PBMCs of asymptomatic adults living in a setting with high incidence of tuberculosis. *M tuberculosis* complex DNA was found in PBMCs of more than three-quarters of participants; detection was more common in CD34-positive than in CD34-negative PBMCs, but it was not restricted to them. Presence of *M tuberculosis* complex DNA in PBMCs was not associated with QuantiFERON status. However, it was more frequent in the presence of HIV infection, and this frequency was nearly halved following administration of isoniazid preventive therapy. By contrast, prevalence of QuantiFERON-positivity was lower in HIV-infected than in HIV-uninfected participants, and it did not change following administration of isoniazid preventive therapy.

Our results are consistent with those of two early reports^8^, ^11^ indicating that *M tuberculosis* complex DNA can be detected in the blood of asymptomatic adults. We extend the findings of these smaller European studies to show that the presence of *M tuberculosis* complex DNA in blood is widespread in a large and heterogeneous cohort of adults living in a setting with high incidence of tuberculosis in Ethiopia. Like Tornack and colleagues,^8^ we found that *M tuberculosis* complex DNA was detected in CD34-positive PBMCs; however, we also detected *M tuberculosis* complex DNA in CD34-negative PBMCs, albeit at a lower frequency and in lower numbers. Of note, we detected *M tuberculosis* complex DNA at considerably higher titre than was reported by Tornack and colleagues in many individuals.^8^ This difference might relate in part to differences in study populations; our study included younger adults living in a tuberculosis-endemic setting with recent exposure or HIV infection in our study, whereas Tornack and colleagues^8^ included older adults living in a low-incidence setting with unknown exposure history. Differences in DNA extraction protocols and in PCR method (dPCR *vs* qPCR) might also explain variation in results of the two studies.

A key difference between our findings and those of Tornack and colleagues^8^ is that we found no association between PCR-positivity and IGRA-positivity. Discordance between results of the QuantiFERON test and our duplex dPCR assay allows discrimination of distinct phenotypes within the latent tuberculosis infection spectrum. It has previously been hypothesised that some individuals might harbour *M tuberculosis* complex infection in the absence of a detectable memory T cell response, whereas others might eliminate *M tuberculosis* complex infection after exposure but retain a memory T cell response.^18^ A priori, one would expect immunocompromised individuals to be over-represented in the former group, and under-represented in the latter. Our observation that HIV infection was more common in QuantiFERON-negative and PCR-positive individuals than in QuantiFERON-positive and PCR-negative individuals is consistent with this expectation and further reinforces the biological plausibility of our findings.

Our study has several strengths. The large blood draw coupled with use of dPCR in duplex format to detect two *M tuberculosis* complex DNA elements—one of them multicopy—confer a high degree of sensitivity. We did rigorous control experiments to exclude cross-contamination or artifact as causes of our positive findings. Use of a prospective study design to show changes in *M tuberculosis* complex copy number following administration of isoniazid preventive therapy provides strong evidence to support our conclusions.

Our study also has some limitations. The absence of extended follow-up for incident active tuberculosis is perhaps the greatest of them and it precludes any comment regarding the prognostic significance of dPCR-positivity in this population. Verma and colleagues reported that two-thirds of initially asymptomatic individuals in whom *M tuberculosis* complex DNA was detected went on to develop active tuberculosis within 7 months of testing.^11^ Given the high prevalence of dPCR-positivity in this study, it seems unlikely that all will progress to active tuberculosis in the short term; taken together with the fact that all participants were asymptomatic, and that none had evidence of active tuberculosis on chest radiography, it would appear that we have detected true latent tuberculosis infection rather than early, presymptomatic, active disease in at least a proportion of those studied here. Future studies could incorporate CT scanning of dPCR-positive individuals to detect radiological evidence of early active tuberculosis with greater sensitivity than a plain chest radiograph can offer. Our results do not allow us to differentiate different members of the *M tuberculosis* complex; whole-genome sequencing approaches are needed to determine whether individuals are infected with *M tuberculosis* or a different member of the *M tuberculosis* complex (such as *M bovis*), and to establish whether infection is with a singe strain or with multilple strains. Heat treatment of samples to kill viable *M tuberculosis* (which allowed us to process them in biosafety level 2 laboratories) precluded attempts at culture. In future work, culture of *M tuberculosis* from PCR-positive cell fractions or indirect detection of viable bacilli indirectly, using the phage method described by Verma and colleagues,^11^ should be attempted. The observation that prevalence of detectable *M tuberculosis* complex DNA was reduced with isoniazid treatment suggests that actively replicating bacteria were present in at least some individuals. These bacteria might reside in the bone marrow or in other cellular or anatomical locations.^7^ Our study was done at a single site, and our findings require validation in other cohorts. It also did not have a dPCR-positive control group that did not receive isoniazid preventive therapy, precluding determination of the spontaneous loss rate. We would also like to highlight that an assay requiring a 100 mL blood draw with PBMC isolation and cell separation before PCR would be challenging to implement at scale, especially in low-income countries. Further work is needed to determine whether the assay can be modified to use smaller volumes of blood or unsorted cells without a loss of sensitivity.

In conclusion, we report some characteristics of a novel molecular microbiological biomarker of latent tuberculosis infection with properties that are distinct from those of a commercial IGRA. Detection of *M tuberculosis* complex DNA in PBMCs has potential applications in the diagnosis of latent tuberculosis infection, in monitoring response to preventive therapy, and as an outcome measure in clinical trials of interventions to prevent or treat latent tuberculosis infection. Longitudinal studies to evaluate the prognostic significance of dPCR-detected *M tuberculosis* complex DNA in PBMCs are now needed, and these will form a major focus of our future work.

## Data sharing

A de-identified copy of the study database will be made available from the corresponding author from the date of publication.

## Declaration of interests

STR has a pending patent that is relevant to the work (WO2017207825A1). All other authors declare no competing interests. The views expressed are those of the authors and not necessarily those of the UK Medical Research Council or the UK Department of Health.
