# Supplementary material for: Detection of Mycobacterium tuberculosis complex DNA in CD34-positive peripheral blood mononuclear cells of asymptomatic tuberculosis contacts: an observational study
Source: Lancet Microbe. 2021 Jun;2(6):e267–75. doi: 10.1016/S2666-5247(21)00043-4 (PMC8172384; doi:10.1016/S2666-5247(21)00043-4)
Supplement: Supplementary appendix [file mmc1.pdf]

THE LANCET
Microbe

Supplementary appendix

This appendix formed part of the original submission and has been peer reviewed. We post it as supplied by the authors.

Supplement to: Belay M, Tulu B, Younis S, et al. Detection of *Mycobacterium tuberculosis* complex DNA in CD34-positive peripheral blood mononuclear cells of asymptomatic tuberculosis contacts: an observational study. *Lancet Microbe* 2021; published online March 31. https://doi.org/10.1016/S2666-5247(21)00043-4.

Detection of *M. tuberculosis* DNA in CD34-positive peripheral blood mononuclear cells of asymptomatic TB contacts: an observational study

Supplementary Appendix

Contents

Title page and Table of Contents ……………………………………………	1
Supplementary Methods …………………………………………………..	2
Table S1. Oligonucleotide information, PCR assays ………..…………….	8
Table S2. Thermal cycling conditions, PCR assays ……..………..………	9
Table S3. dMIQE checklist ……………………………………………….	10
Table S4. Detection of IS*6110* in CD34-positive vs. CD34-negative peripheral blood mononuclear cells of asymptomatic adults ……………..	13
Table S5. Detection of *rpoB* in CD34-positive vs. CD34-negative peripheral blood mononuclear cells of asymptomatic adults ……………..	13
Figure S1. Flow cytometry plots showing surface expression of CD34 in positively- and negatively-selected PBMC populations …………………..	14
Figure S2. Representative dPCR amplification plots from PBMC fractions with multiple positive droplets……………………………………...	15
Figure S3. Representative dPCR amplification plots from PBMC fractions with very low numbers of positive droplets ……..…………….	16
Figure S4. Representative dPCR amplification plots from PBMC fractions without positive droplets ……………………………………………	17
Figure S5. dPCR amplification plots for extracts of *M. tuberculosis*-infected monocyte-derived macrophages…………………………………...	18
Figure S6. MTBC gene copy number per 20 µl well for a dilution series of *M. tuberculosis* DNA …………………………………………………….	19
Figure S7. Control experiments ………………………………………………	20
Figure S8. MTBC gene copy number per 20 µl well vs antigen-stimulated IFN-γ ……...	21
Figure S9. dPCR status by HIV viral load ……………………………….	22
Figure S10. dPCR status by previous history of active TB ………………	23
References ………………………………………………………………	24

Supplementary Methods

Interferon-γ Release Assay

1 ml whole blood was drawn into each of four QuantiFERON-TB Gold Plus tubes (Qiagen, Hilden, Germany), which were gently inverted ten times prior to incubation at 37ºC for 18 hours. Tubes were then centrifuged at 2000g for 15 minutes, and plasma supernatants were aspirated and frozen at -80ºC pending determination of IFN-γ concentrations by ELISA, performed according to manufacturer's instructions. Results were calculated using QuantiFERON-TB Gold Plus Analysis Software (Qiagen).

PBMC isolation

PBMC were isolated in a Biosafety Level 2 laboratory in Addis Ababa that had never been used for work with MTBC organisms. For each donor, 100 ml peripheral blood were diluted with 110 ml phosphate buffered saline (PBS) without calcium or magnesium (Sigma-Aldrich, St Louis, MO). Thirty-five ml aliquots of diluted blood were then layered onto 15 ml HistoPaque-1077 (Sigma-Aldrich) in each of six Leucosep tubes (Greiner Bio-One GmbH, Kremsmünster, Austria), which were then centrifuged at 1000g for 15 minutes, without braking at the end of the spin. Plasma supernatants were removed from each tube, and PBMC layers were transferred equally into four Falcon 50 ml centrifuge tubes (ThermoFisher Scientific, Waltham, MA), which were then topped up to a volume of 50 ml each, using cold MACS buffer (0.5% bovine serum albumin and 2mM EDTA [both from Sigma-Aldrich] in PBS). These tubes were then centrifuged at 300g for 10 minutes, with braking at the end of the spin. Supernatants were discarded, and cell pellets were re-suspended and pooled into a single 50 ml centrifuge tube, which was topped up to 50 ml with MACS buffer and centrifuged at 200g for 10 minutes, with braking at the end of the spin. The supernatant was discarded, and cell pellets were re-suspended and counted. The mean PBMC yield per donor was 153.5 million cells (standard deviation 58.4 million cells); mean purity (i.e. the proportion of cells that were not granulocytes) was 90.0% (standard deviation 3.5%).

Separation of CD34-positive vs. CD34-negative PBMC

Following counting, PBMCs were topped up to 30mL with MACS buffer, and centrifuged at 200g for 10 minutes at 4ºC, with braking at the end of the spin. The supernatant was aspirated, and the cell pellet was re-suspended in 300 µL of MACS buffer for up to 10^8 total

cells; where more than 10^8 total cells were isolated, reagents in the steps below were scaled up in proportion. 100 µL of FcR blocking reagent (Miltenyi Biotec, Bergisch Gladbach, Germany) was added to the cell suspension, prior to further addition of 100 µl of human CD34 UltraPure MicroBeads (Miltenyi). Solutions were mixed well, and incubated for 30 minutes at +4ºC. Cells were then washed by adding 10 mL of MACS buffer and centrifuging at 300g for 10 minutes at +4ºC, with braking at the end of the spin. The supernatant was aspirated, and the cell pellet was re-suspended in 500 µL of MACS buffer and applied via a 30 µm filter (Miltenyi) to a MS separation column ('MS column #1') that had been pre-rinsed with 500 µL of MACS buffer and placed in the magnetic field of a Mini-MACS separator (Miltenyi). The flow-through from MS column #1 was collected. MS column #1 was then washed with 3×500µL MACS buffer. The flow-through from each wash was pooled with the initial flow-through, and the total flow-through from MS column #1 was then applied to a second pre-rinsed MS separation column (MS column #2) in a Mini-MACS separator, with the flow-through collected as before. MS column #2 was then washed with 3×500µL MACS buffer, and the flow-through from each wash was pooled with the initial flow-through from MS column #2 to comprise the final suspension of CD34-negative cells. MS columns #1 and #2 were removed from the magnetic separator, and positively selected magnetically labelled cells were flushed out of each column using a plunger with 1 ml MACS buffer and pooled to comprise the final suspension of CD34-positive cells. Finally, tubes containing each fraction were centrifuged at 500g for 10 minutes, with braking at the end of the spin. Supernatants were aspirated, and CD34-positive and –negative cell pellets were heated in a water bath to 80ºC for 20 minutes to render them sterile prior to immediate freezing at -80ºC pending DNA extraction. The number of cells in the CD34-positive fraction was not counted, as it was too small to enumerate with any accuracy using a counting chamber at working dilution.

Flow cytometry

Effective enrichment / depletion of CD34-positive PBMC from positively / negatively selected cell fractions was confirmed by flow cytometry in one donor. Staining for surface expression of CD34 and CD45 on live PBMC was performed according to standard protocols using PE-conjugated anti-human CD34 and Pacific Blue™-conjugated anti-human CD45 (both from Biolegend, San Diego, CA). PBMC were gated on the basis of forward / side scatter, and live cells were gated on the basis of low staining with the Zombie NIR dye (Biolegend). CD34+/CD45+ cells constituted 0.14% of unselected live PBMC prior to magnetic bead

separation; 0.006% of live PBMC in the negatively-selected cell fraction; and 71.6% of live PBMC in the positively-selected cell fraction (Fig. S1).

DNA extraction

DNA was extracted within 12 months of blood sample collection in a Biosafety Level 2 laboratory in London that had never been used for work with MTBC organisms, using a modified version of a protocol by van Soolingen et al.[1] Thawed cell pellets were incubated with 50 µL of 10 mg/mL lysozyme (Sigma-Aldrich, Gillingham, UK) for 5 hours at 37ºC prior to addition of 10 µL of 20 mg/mL proteinase K (Qiagen), 10 µL of 10% sodium dodecyl sulphate (SDS, Sigma-Aldrich) and 180 µL H_2O and extension of incubation at 37ºC overnight. The following morning, the entire volume of the digested CD34-positive pellet and a 10% aliquot of the digested CD34-negative pellet were made up to 450 µL with H_2O prior to addition of 70 µL 10% SDS and 100 µL 5M NaCl (Sigma-Aldrich) with gentle mixing prior to the further addition of 100 µL of pre-warmed Cetyltrimethylammonium bromide (CTAB)/NaCl buffer that had been previously prepared by dissolving 4.1g NaCl and 10g CTAB 99+% (ThermoFisher Scientific, Waltham, MA) in 100 ml H_2O. This mixture was then incubated at 65°C for 10 minutes, prior to addition of 750 µL chloroform/isoamyl alcohol (24:1 v/v, VWR, Radnor, PA) to each tube followed by gentle mixing to achieve complete dispersal of the sample. Samples were then centrifuged at 10,000g for 5 minutes prior to transfer to a fume hood and careful transfer of the upper (aqueous) supernatant layers into new vials, to which 450 µL of cold (-20°C) 100% isopropanol (Sigma-Aldrich) was then added. Tubes were gently inverted and chilled at -20°C for 30 minutes prior to centrifugation at 10,000g for 15 minutes at ambient temperature. Supernatants were then discarded, and pellets were washed with 1 mL of cold (-20°C) 70% ethanol (Sigma-Aldrich) prior to centrifugation at 10,000g for 5 minutes at ambient temperature. The supernatant was then aspirated, and pellets were heated to dryness in a heating block (65ºC) prior to rehydration in 0.1xTris-EDTA buffer (TE buffer, Sigma-Aldrich; 33 µl for CD34-positive pellets, 50 µl for CD34-negative pellets) at 4ºC overnight. Concentrations of DNA were determined using the NanoDrop 8000 spectrophotometer (ThermoFisher Scientific): average DNA concentrations extracted from CD34-positive vs –negative cell pellets were 369.8 vs. 836.5 ng/µl.

Effectiveness of this protocol for extraction of DNA from intracellular *M. tuberculosis* was confirmed as follows. 10^6 monocyte-derived macrophages were infected with $5x10^6$ colony-forming units of *M. tuberculosis* H37Rv. After a 4-hour incubation, cells were washed three

times with warm phosphate-buffered saline to remove non-phagocytosed bacilli. Infected cells were then heated to 80ºC for 30 minutes, and DNA was extracted from heat-treated material as described above. dPCR for IS*6110* and *rpoB* was performed on 1:100 dilutions of extracts in duplicate: DNA of both genes was readily detectable (Fig S5), providing confirmation that the CTAB protocol was effective for extraction of DNA from intra-cellular *M. tuberculosis*.

Digital PCR

dPCR was performed on samples within 6 months of DNA extraction. Prior to dPCR, solutions of DNA extracted from CD34-negative cell pellets were diluted so that their DNA concentration matched that of the corresponding solution of DNA extracted from CD34-positive cell pellets of that donor. A duplex dPCR assay was performed in all samples using the QX200 AutoDG Droplet Digital PCR System (Bio-Rad Laboratories, Hercules CA) to detect DNA of two MTBC genes that are absent from non-tuberculous mycobacteria: insertion sequence (IS)*6110*, a multi-copy gene that was utilised to maximise assay sensitivity, and *rpoB*, a single-copy gene that may undergo mutations that confer rifampicin resistance. Singleplex assays for *csrR* (*Streptococcus pyogenes*), *coA* (*Staphylococcus aureus*) and *femA_SE* (*Staphylococcus epidermidis*) were also performed on a subset of 20 samples (10 in which DNA for both IS*6110* and *rpoB* had been detected with dPCR, and 10 in which DNA of neither gene had been detected). Details of HPLC-purified oligonucleotides used in each assay (supplied by Sigma-Aldrich) are presented in Table S1. 22 µL reactions were prepared for analysis containing 10 µL of sample extract, 11µl 2× dPCR Supermix for Probes (Bio-Rad) and 1 µL of 22× primer/probe mix, and pipetted into a 96-well dPCR plate (Bio-Rad). 40 µL of droplets per well were generated using the autoDG QX200 Droplet Generator according to the manufacturer's instructions. 96-well plates were sealed with foil using the Bio-Rad PX1 PCR plate sealer and PCR was performed using the Bio-Rad C1000 touch thermal cycler; thermal cycling conditions are presented in Table S2. Droplets were analysed using the QX200 droplet reader according to manufacturer's instructions. Data were analysed using QuantaSoft™ Analysis Pro software version 1.0.596 and thresholds were set at amplitudes of 3000 for channel 1 (detecting the FAM-labelled *rpoB* probe) and at 1500 for channel 2 (detecting the HEX-labelled IS*6110* probe) across all the samples to separate the positive and negative droplets for each assay. Representative amplification plots of positive and negative clinical samples are presented in Figs. S2-S4. Data from wells with droplet counts <10,000 were excluded from statistical analyses, according to the manufacturer's

instructions. Mean droplet count from wells with droplet counts >10,000 was 16,725 (standard deviation 2,983, range 10,002 to 27,190). Assuming the manufacturer's published volume of 0.85 nL per droplet, the mean total volume of the droplets measured per reaction was 14.2 µl (standard deviation 2.5 µl, range 8.5 to 23.1 µl).

Control experiments

We conducted several control experiments and analyses to investigate cross-contamination with *M. tuberculosis* DNA / amplicons and/or non-specific amplification as causes of false-positive results. In order to investigate whether amplicon contamination could explain our positive results, we sent 10 clinical samples that were positive for both *rpoB* and IS*6110* with our dPCR assay for detection of a different MTBC target (*ESAT6*) using qPCR in another investigator's laboratory (Molecular Bacteriology Laboratory, Great Ormond St Hospital for Children, London). Cross-contamination from a laboratory source in Ethiopia was investigated by dPCR analysis of triplicate swabs of benches and microbiological safety cabinets in the laboratory where blood samples were processed. If cross-contamination had occurred in the laboratory in Addis Ababa, one might expect PCR-positive samples to be clustered in time, e.g. if a particular batch of reagent that was in use for a certain period had become contaminated. To check for this, we plotted copy number for IS*6110* and *rpoB* over the course of the study.

Non-specific amplification of sequences present in host DNA was investigated by analysis of 32 replicates of a reference human DNA sample (Centre d'Etude du Polymorphism Humain, Paris, France). To explore the possibility of non-specific amplification of sequences present in organisms other than MTBC, we conducted specificity controls using DNA from non-tuberculous mycobacteria (*M. abscessus* and *M. szulgai*) and some common skin commensals (*Staphylococcus epidermidis*, *Staphylococcus aureus* and *Streptococcus pyogenes*). We also ran dPCR on extracts from 20 donors (10 in which DNA for both IS*6110* and *rpoB* had been detected with dPCR, and 10 in which DNA of neither gene had been detected) for targets in *S. pyogenes (csrR), S.aureus (coA)* and *S. epidermidis* (*femA_SE*). False-positive dPCR results arising due to magnetic bead artefact were investigated by analysis of PBMC samples into which 50 µL (n=5 samples) and 100 µL (n=5 samples) of UltraPure MicroBeads (Miltenyi) had been spiked.

Quantitative PCR

A qPCR assay for *ESAT6* (MTBC) was performed on the ABI 7500 RealTime PCR System (Applied BioSystems) at Great Ormond Street Hospital, London, UK, employing oligonucleotides and thermal cycling conditions as described in Tables S1 and S2, respectively. Data were analysed using ABI 7500 System software version 1.4.1.

M. tuberculosis gDNA dilution series

Genomic DNA extracted from a *M. tuberculosis* strain of East Asian / Beijing lineage (VNTR 42435 2332515333 456443382) was obtained from the National Mycobacterium Reference Laboratory, Colindale, London, UK, reconstituted in nuclease-free water and stored at -80°C. Serial 10-fold dilutions of this material ranging from 2000 copies/reaction to 2 copies/reaction were assayed with the duplex dPCR assay for IS*6110* and *rpoB* as described above (Fig. S6).

Blinding of laboratory staff

Laboratory staff who isolated PBMCs and who performed PCR were blinded to QFT status. Clinical samples sent for qPCR to Great Ormond Street Hospital were blinded, i.e. they were labelled with a numeric identifier that did not indicate whether they were dPCR-positive or dPCR-negative samples.

Table S1. Oligonucleotide information, PCR assays

Assay name	Oligonucleotide name	Oligo sequence	Reporter, Quencher	[oligos], nM	Amplicon length, bp	Reference
IS*6110*[(1)]	IS6110_F	AGAAGGCGTACTCGACCTGA	-	100	157	This paper
	IS6110_R	GATCGTCTCGGCTAGTGCAT	-	100		
	IS6110_P	AGGCAGGCATCCAACCG	HEX, BHQ1	250		
rpoB[(2)]	rpoB_F	CAAAACAGCCGCTAGTCCTAGTC	-	900	84	[2]
	rpoB_R	AAGGAGACCCGGTTTGGC	-	900		
	rpoB_P	AGTCGCCCGCAAAGTTCCTCGAA	FAM, BHQ1	250		
ESAT6[(3)]	ESAT6_F	GGAATTTCGCGGGTATCGA	-	800	65	This paper
	ESAT6_R	GGAATGAATGGACGTGACATTTC	-	800		
	ESAT6_P	CGCGGCAAGCGCAATCCA	JOE, BHQ1	800		
csrR[(4)]	csrR_F	TGGATGTGGTTGCAGGTTTAGAC	-	900	79	[3]
	csrR_R	CGGGCAAGTAGTTCTTCAATGG	-	900		
	csrR_P	CGGTGCAGACGACTATATTGTTAAACC	FAM, BHQ1	250		
coA[(5)]	coA_F	GTAGATTGGGCAATTACATTTTGGAGG	-	900	117	[3]
	coA_R	CGCATCTGCTTTGTTATCCCATGTA	-	900		
	coA_P	TAGGCGCATTAGCAGTTGCATC	HEX, BHQ1	250		
femA_SE[(6)]	femA_SE_F	TGCAGGGAGCTATGCGGTTCAATG	-	900	119	This paper
	femA_SE_R	CGCCAGCATCTTCAGCATCTTCAC	-	900		
	femA_SE_P	CCATGTTCAATTGCATAGTTAATCATCT	HEX, BHQ1	250		

1, the locations of the IS*6110* assay are 889985...890141, 1542342...1542186, 1988093...1987937, 1997065...1997221, 2366378...2366534, 2430507...2430351, 2550978...2551134, 2636541...2636697, 2785005...2784849, 2973073...2973229, 3120914...3120758, 3552194...3552350, 3553677...3553833, 3711346...3711502, 3795448...3795292, 3891743...3891899. Sequence information based on *Mycobacterium tuberculosis* H37Rv, NC_018143.

2, the location of the *rpoB* assay is 759827… 759910. Sequence information based on *Mycobacterium tuberculosis* H37Rv, NC_018143.

3, the location of the *ESAT6* assay is 4352803 ... 4352867. Sequence information based on *Mycobacterium tuberculosis* H37Rv, NC_018143.

4, the location of the *csrR* assay is 294165 … 294243. Sequence information based on S*treptococcus pyogenes* strain AUSMDU00010539, accession number CP045930.1

5, the location of the *coA* assay is 2423 … 2539. Sequence information based on *Staphylococcus aureus* strain NVAU02081, accession number AB436985.

6, the location of the *femA_SE* assay is 1398 … 1516. Sequence information based on *Staphylococcus epidermidis*, accession number U23713.1.

Table S2. Thermal cycling conditions, PCR assays

Assay name	Mastermix (Manufacturer)	Enzyme activation	Denaturation	Primer annealing and extension	Droplet stabilisation	Maintenance	Ramp rate
IS*6110*, *rpoB*	dPCR: 2X dPCR SuperMix for probes (BioRad)	95ºC, 600s	95ºC, 30s	55ºC, 60s	95ºC, 600s	4ºC	2.5ºC/s
ESAT6	2X QuantiFast Multiplex PCR Mastermix (Qiagen)	95ºC, 600s	95ºC, 30s	60ºC, 30s	NA	NA	NA
csrR, coA, femA_SE	2X dPCR SuperMix for probes (BioRad)	95ºC, 600s	94ºC, 30s	60ºC, 60s	98ºC, 600s	4ºC	2.0ºC/s

Table S3. dMIQE checklist

ITEM	WHERE REPORTED
**EXPERIMENTAL DESIGN**	
Definition of experimental and control groups	Methods, Main Manuscript
Number within each group	Methods, Main Manuscript
Assay carried out by core lab or investigator's lab?	Methods, Supplementary Appendix
Power calculation	Methods, Supplementary Appendix
**SAMPLE**	
Description	Methods, Main Manuscript and Supplementary Appendix
Volume/mass of sample processed	Methods, Main Manuscript
Microdissection or macrodissection	N/A
Processing procedure	Methods, Main Manuscript and Supplementary Appendix
If frozen - how and how	Methods, Supplementary Appendix
If fixed - with what, how	N/A
Sample storage conditions and duration (especially for FFPE samples)	Methods, Supplementary Appendix
**NUCLEIC ACID EXTRACTION**	
Nucleic acid quantification	Methods, Supplementary Appendix
DNA or RNA quantification	DNA
Quality/Integrity,	Methods, Supplementary Appendix
Template structural information	Methods, Supplementary Appendix
Template modification (digestion, sonication, preamplification etc.)	Methods, Supplementary Appendix
Template treatment	None
Inhibition dilutions or spike	Methods and Fig. S6, Supplementary Appendix
DNA contamination assessment of RNA samples	N/A
Details of DNase treatment where performed	N/A
Manufacturer of reagents used and catalogue number	Methods, Main Manuscript and Supplementary Appendix
Storage conditions (Nucleic acid): temperature, concentration, duration, buffer)	Methods, Main Manuscript and Supplementary Appendix
**dPCR TARGET INFORMATION**	
Sequence accession number	Table S1, Supplementary Appendix
Location of amplicon	Table S1, Supplementary Appendix
Amplicon length	Table S1, Supplementary Appendix
In silico specificity screen (BLAST, etc.)	NCBI PrimerBlast

ITEM (continued)	WHERE REPORTED (continued)
Pseudogenes, retropseudogenes or other homologs?	N/D
Sequence alignment	Available upon request
Secondary structure analysis of amplicon and GC content	Available upon request
Location of each primer by exon or intron (if applicable)	N/A (bacterial DNA)
What splice variants are targeted?	N/A (bacterial DNA)
**dPCR OLIGONUCLEOTIDES**	
Primer sequences	Table S1, Supplementary Appendix
RTPrimerDB Identification Number	N/A
Probe sequences	Table S1, Supplementary Appendix
Location and identity of any modifications	N/A
Manufacturer of oligonucleotides	Methods, Supplementary Appendix
Purification method	Methods, Supplementary Appendix
Complete reaction conditions	Methods & Table S1, Supplementary Appendix
Reaction volume and amount of cDNA/DNA	Methods, Supplementary Appendix
Primer, (probe), Mg++ and dNTP concentrations	Methods & Table S1, Supplementary Appendix
Polymerase identity and concentration	Methods, Supplementary Appendix
Buffer/kit identity and manufacturer	Methods, Supplementary Appendix
Exact chemical constitution of the buffer	Proprietary
Additives (SYBR Green I, DMSO, etc.)	No additives
Plates/tubes catalogue number	Available upon request
Complete thermocycling	Table S2, Supplementary Appendix
Reaction setup (manual/robotic)	Manual
Gravimetric or volumetric dilutions (manual/robotic)	Dilution of template: manual volumetric. Addition of sample to dPCR reaction mix: manual volumetric.
Total PCR volume prepared	Methods, Supplementary Appendix
Partition number	Methods, Supplementary Appendix
Individual partition volume	Methods, Supplementary Appendix
Total volume of the partitions measured (effective reaction size)	Methods, Supplementary Appendix
Partition volume variance/SD	N/D
Comprehensive details and appropriate use of controls	Methods, Supplementary Appendix
Manufacturer of dPCR	Methods, Supplementary Appendix
Optimization data for the assay	Available upon request

ITEM (continued)	WHERE REPORTED (continued)
Specificity (when measuring rare mutations, pathogen sequences etc.)	N/A
Limit of detection of calibration	N/A
If multiplexing, comparison with singleplex assays	Available upon request
**DATA ANALYSIS**	
Mean copies per partition (λ or equivalent)	<0.01
dPCR analysis program (source, version)	Methods, Supplementary Appendix
Outlier identification and disposition	No statistical outliers were removed
Results of NTCs	Methods, Supplementary Appendix, Figs S2, S3, S4, S5
Examples of positive(s) and negative experimental results as supplemental data	Figs S2-S4
Where appropriate, justification of number and choice of reference genes	N/A
Where appropriate, description of normalization method	N/A
Number and concordance of biological replicates	Available upon request
Number and stage (RT or qPCR) of technical replicates	N/A
Repeatability (intra-assay variation)	Available upon request
Reproducibility (inter-assay/user/lab etc. variation)	Available upon request
Experimental variance or CI	Available upon request
Statistical methods for analysis	Methods, Main Manuscript
Data submission using RDML (Real-time PCR Data Markup Language)	Available upon request

Table S4. Detection of IS*6110* in CD34-positive vs. CD34-negative peripheral blood mononuclear cells of asymptomatic adults. P <0.0001, Fisher's exact test.

		CD34-positive	
		IS*6110* undetected	IS*6110* detected
CD34-negative	IS*6110* undetected	48	105
	IS*6110* detected	2	42

Table S5. Detection of *rpoB* in CD34-positive vs. CD34-negative peripheral blood mononuclear cells of asymptomatic adults. P =0.0031, Fisher's exact test.

		CD34-positive	
		rpoB undetected	*rpoB* detected
CD34-negative	*rpoB* undetected	104	70
	rpoB detected	6	17

Figure S1. Flow cytometry plots showing surface expression of CD34 in positively- and negatively-selected PBMC populations. Staining was performed using PE-conjugated anti-human CD34 and Pacific Blue^{TM}-conjugated anti-human CD45 (Biolegend, San Diego, CA). A single test was performed for each fraction to confirm manufacturer-reported performance. **A**, unselected PBMC prior to magnetic bead separation. **B**, negatively selected cells (passive flow-through from MS columns). **C**, positively-selected cells (actively flushed from MS columns following removal of column from magnetic separator). PBMC were gated on the basis of forward / side scatter, and live cells were gated on the basis of low staining with the Zombie NIR dye (Biolegend).

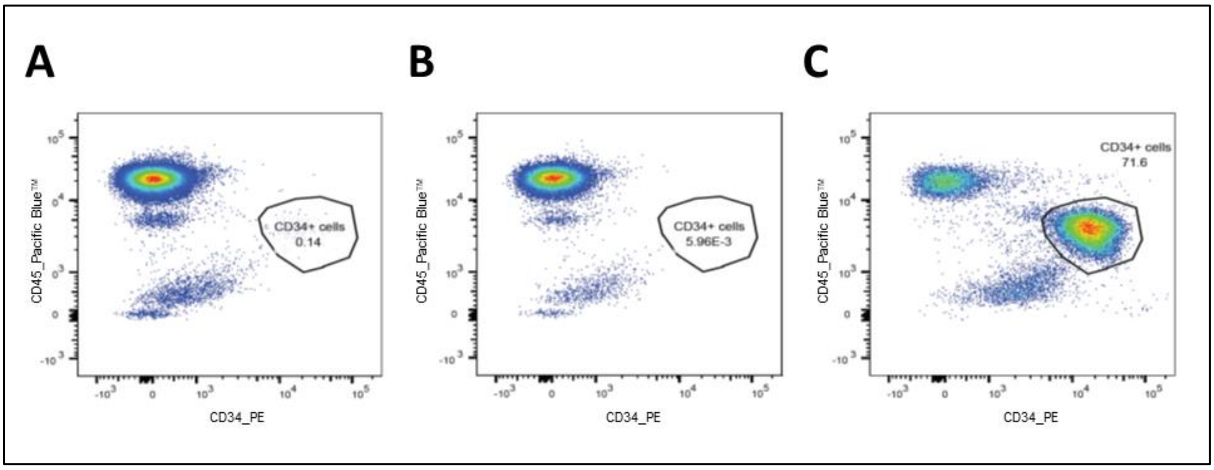

Figure S2. dPCR amplification plots for extracts of *M. tuberculosis*-infected monocyte-derived macrophages. 10^6 monocyte-derived macrophages (MDM) were infected with $5x10^6$ colony-forming units of *M. tuberculosis* H37Rv. After a 4-hour incubation, cells were washed three times with warm phosphate-buffered saline to remove non-phagocytosed bacilli. Infected cells were then heated to 80ºC for 30 minutes, DNA was extracted as described in Supplementary Methods, and dPCR for rpoB (A) and IS6110 (B) was performed on 1:100 dilutions of extracts. Amplification plots for uninfected MDM, *M. tuberculosis* DNA and non-template control (NTC) are also shown.

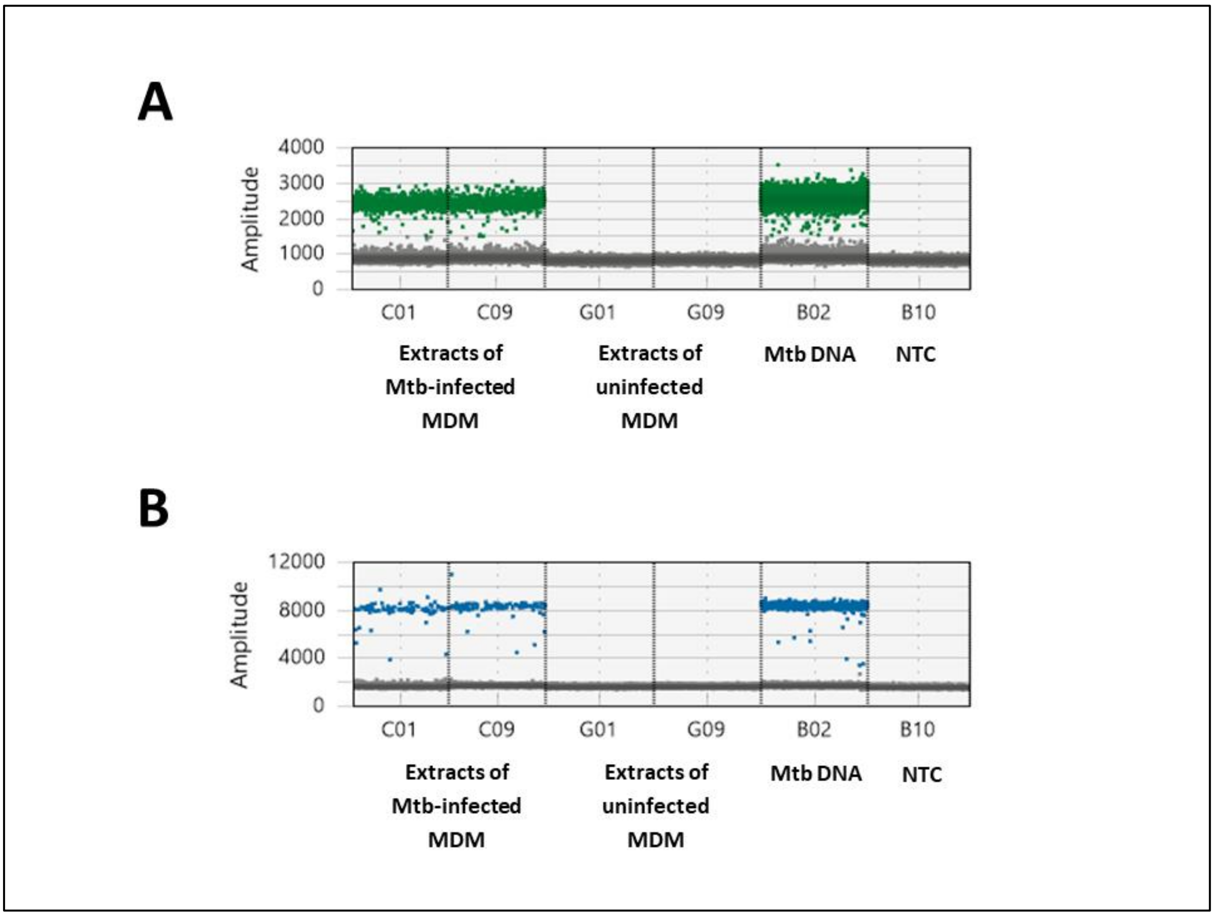

Figure S3. MTBC gene copy number per 20 µl well for a dilution series of *M. tuberculosis* DNA. A, IS*6110*; **B**, *rpoB*. *M. tuberculosis* DNA was spiked into hDNA background at 150 ng/well in quantities ranging from 2 copies per reaction to 2000 copies per reaction.

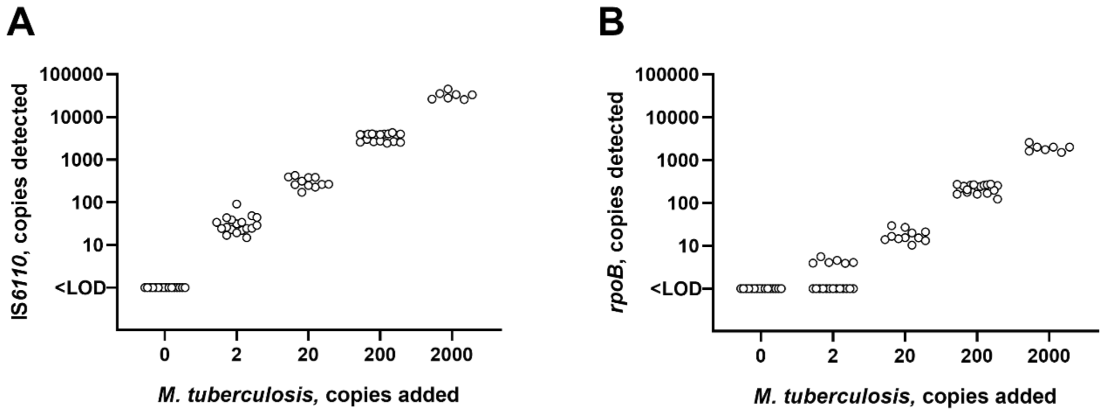

Figure S4. Representative dPCR amplification plots from PBMC fractions with multiple positive droplets (n=6). A, for *rpoB* (FAM-labelled probe, channel 1). **B**, for IS*6110* (HEX-labelled probe, channel 2). P1-6 are participant samples; NTC, non-template control. POS, positive control (100 fg *M. tuberculosis* DNA). Pink lines show thresholds set at amplitudes of 3000 and 1500 for channels 1 and 2, respectively.

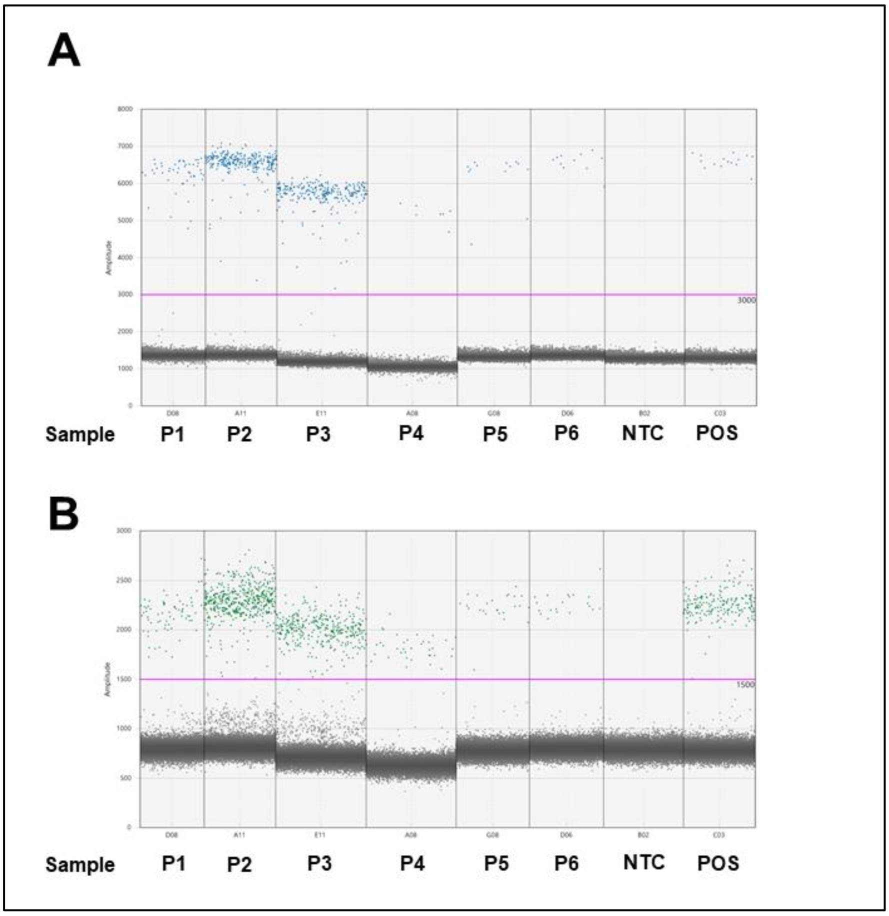

Figure S5. Representative dPCR amplification plots from PBMC fractions with very low numbers of positive droplets (n=6). These samples were called as dPCR-negative on the basis that the calculated copy number per 20 µl well was below limits of detection, calculated as 4.38 and 3.80 copies per 20 µl well for IS*6110* and *rpoB*, respectively. **A**, for *rpoB* (FAM-labelled probe, channel 1). **B**, for IS*6110* (HEX-labelled probe, channel 2). P7-12 are participant samples; NTC, non-template control. POS, positive control (100 fg *M. tuberculosis* DNA). Pink lines show thresholds set at amplitudes of 3000 and 1500 for channels 1 and 2, respectively.

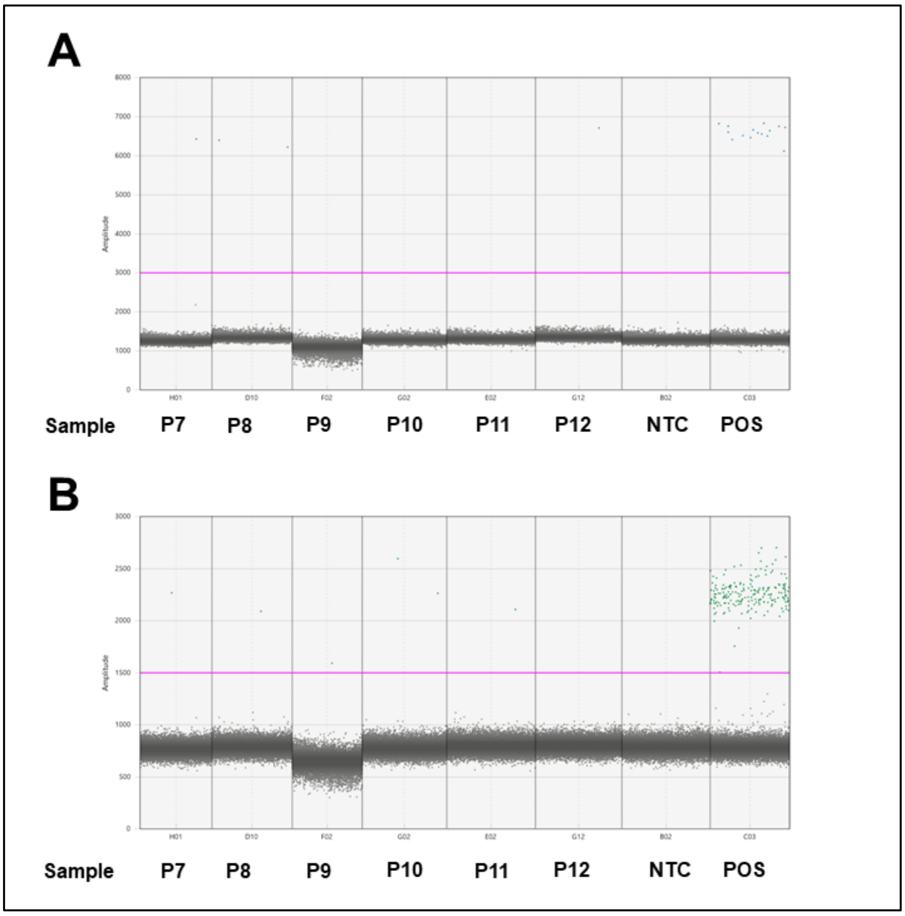

Figure S6. Representative dPCR amplification plots from PBMC fractions without positive droplets (n=6). A, for *rpoB* (FAM-labelled probe, channel 1). **B**, for IS*6110* (HEX-labelled probe, channel 2). P13-18 are participant samples; NTC, non-template control. POS, positive control (100 fg *M. tuberculosis* DNA). Pink lines show thresholds set at amplitudes of 3000 and 1500 for channels 1 and 2, respectively.

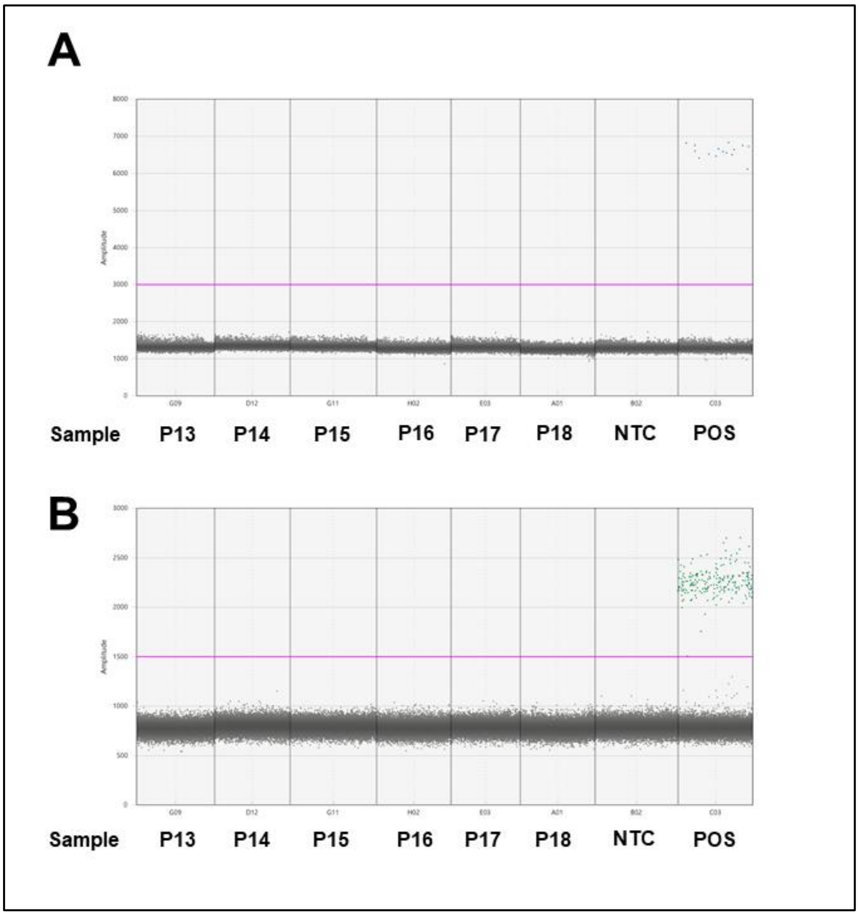

Figure S7. Control experiments. A, amplification plot from qPCR assay for *ESAT6* of 10 clinical samples in which DNA for both IS*6110* and *rpoB* had been detected using dPCR. **B**, cycle threshold for *ESAT6* (qPCR) vs. copy no. for *rpoB* (dPCR) in the same 10 samples. R and P values, Spearman's rho. **C.** IS*6110* gene copy number per 20 µl well over the course of the study. **D**, *rpoB* gene copy number per 20 µl well over the course of the study. Study day, number of days elapsed since recruitment of first participant. LOD, limit of detection.

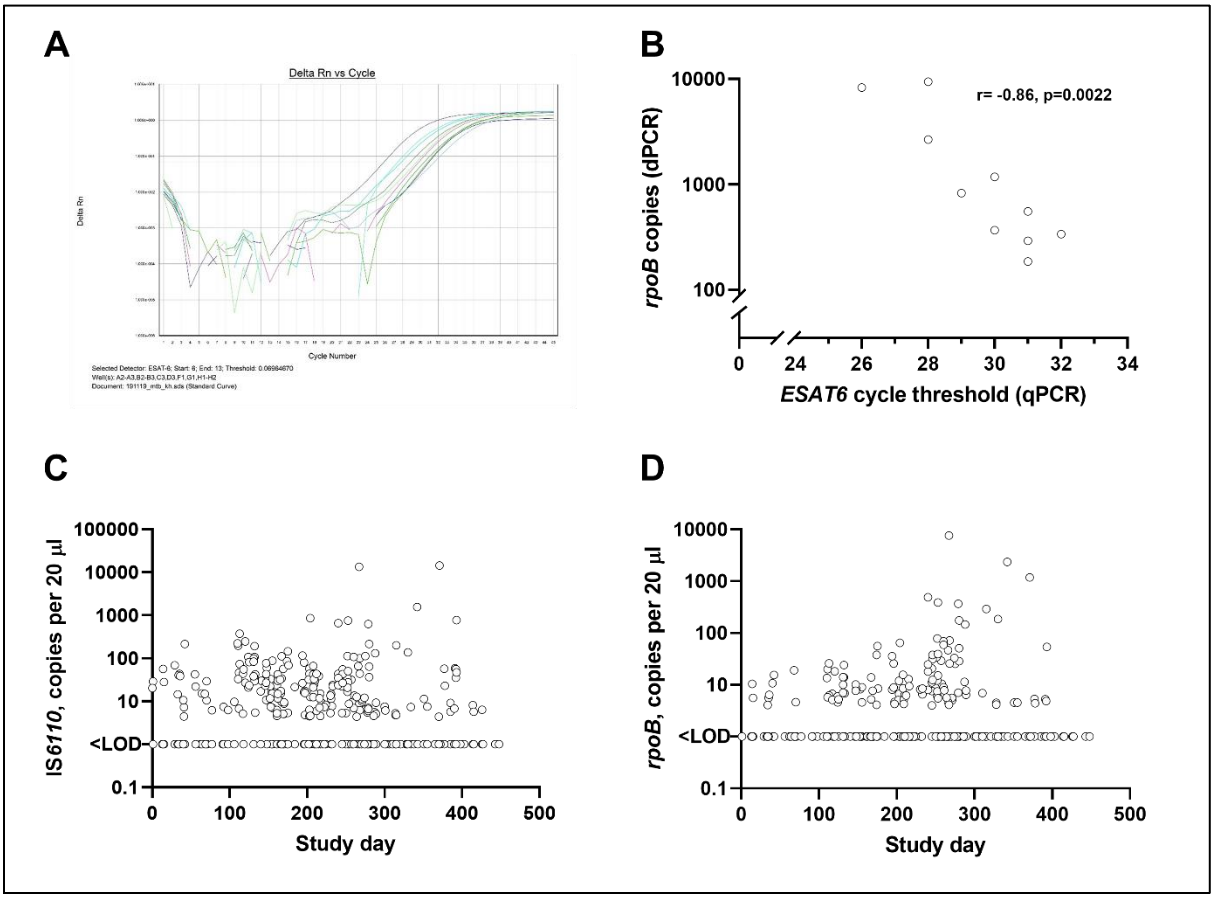

Figure S8: MTBC gene copy number per 20 µl well vs antigen-stimulated IFN-γ. A, IS*6110* copy no. vs TB1-stimulated IFN-γ, CD34-negative PBMC. **B**, IS*6110* copy no. vs TB2-stimulated IFN-γ, CD34-negative PBMC. **C**, IS*6110* copy no. vs TB1-stimulated IFN-γ, CD34-positive PBMC. **D**, IS*6110* copy no. vs TB2-stimulated IFN-γ, CD34-positive PBMC. **E**, *rpoB* copy no. vs TB1-stimulated IFN-γ, CD34-negative PBMC. **F**, *rpoB* copy no. vs TB2-stimulated IFN-γ, CD34-negative PBMC. **G**, *rpoB* copy no. vs TB1-stimulated IFN-γ, CD34-positive PBMC. **H**, *rpoB* copy no. vs TB2-stimulated IFN-γ, CD34-positive PBMC.

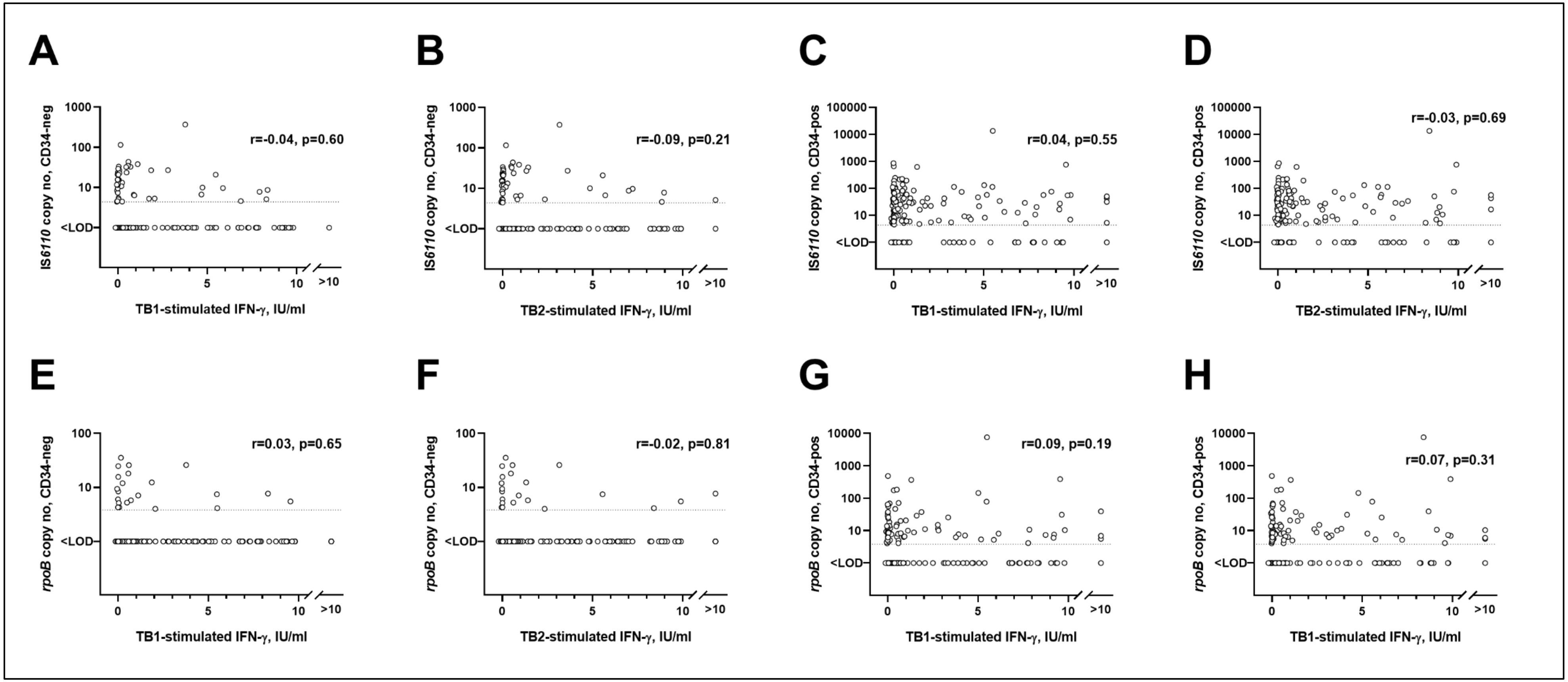

Figure S9: dPCR status by HIV viral load. A, PCR status by HIV viral load; P value from Fisher's exact test. **B**, IS*6110* copy number per 20 µl well by HIV viral load in CD34-negative and CD34-positive PBMC; P values from Mann Whitney tests, lines show median values. Proportions with undetectable MTBC DNA are also displayed. **C**, *rpoB* copy number per 20 µl well by HIV viral load in CD34-negative and CD34-positive PBMC; P values from Mann Whitney tests, lines show median values. Proportions with undetectable MTBC DNA are also displayed. LOD, limit of detection.

A

		**MTBC DNA**	
		Undetected	Detected
**HIV viral load**	0	4/64 (6.3%)	55/64 (85.9%)
	>0	2/64 (3.1%)	3/64 (4.7%)

p=0.065

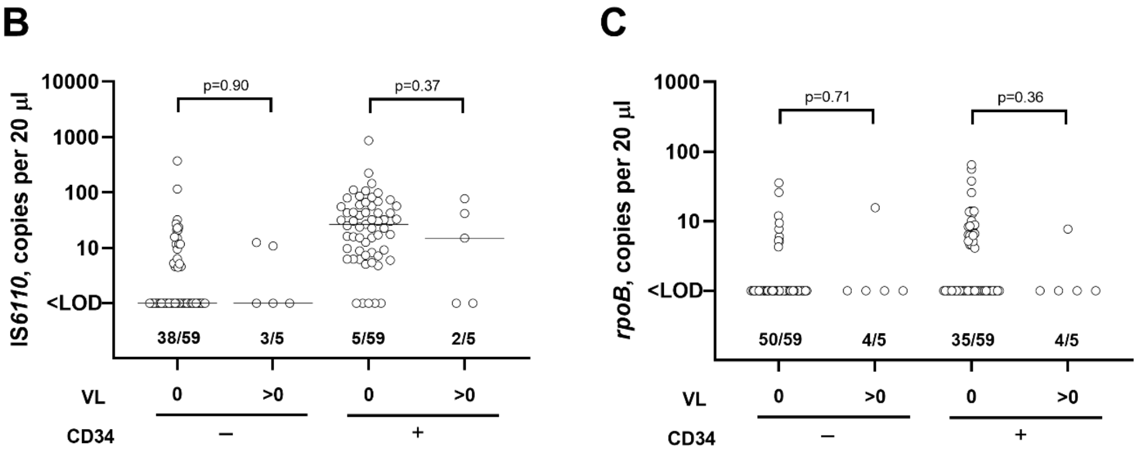

Figure S10: dPCR status for participants with vs. without previous history of active TB. A, PCR status by previous active TB; P value from Fisher's exact test. **B**, IS*6110* copy number per 20 µl well by previous active TB in CD34-negative and CD34-positive PBMC; P values from Mann Whitney tests, lines show median values. Proportions with undetectable MTBC DNA are also displayed. **C**, *rpoB* copy number per 20 µl well by previous active TB in CD34-negative and CD34-positive PBMC; P values from Mann Whitney tests, lines show median values. Proportions with undetectable MTBC DNA are also displayed. LOD, limit of detection.

A

		**MTBC DNA**	
		Undetected	Detected
**Previous TB**	No	5/72 (6.9%)	53/72 (73.6%)
	Yes	3/72 (4.2%)	11/72 (15.3%)

p=0.18

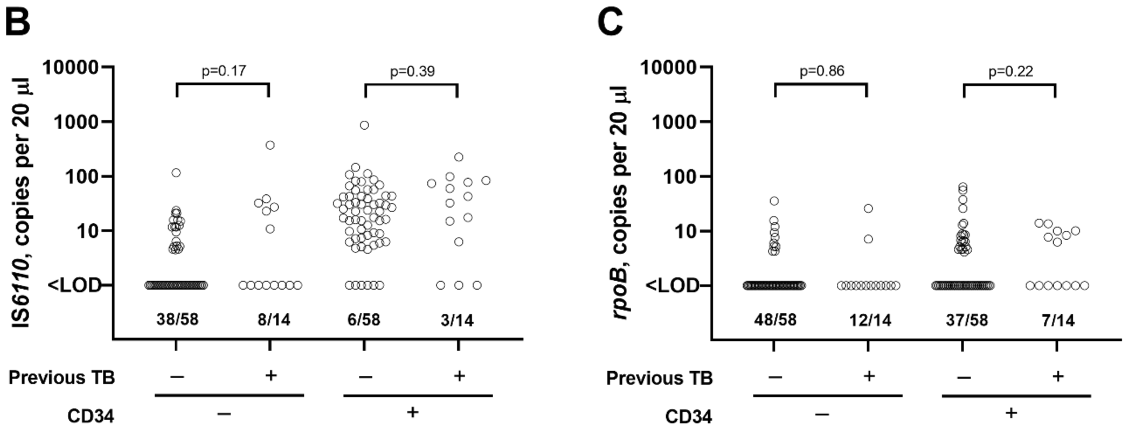

REFERENCES

1. van Soolingen D, de Haas PEW, Kremer K. Restriction Fragment Length Polymorphism Typing of Mycobacteria. In: Parish T, Stoker NG, eds. *Mycobacterium tuberculosis* Protocols. Totowa, NJ: Humana Press; 2001.
2. Devonshire AS, Honeyborne I, Gutteridge A, et al. Highly reproducible absolute quantification of Mycobacterium tuberculosis complex by digital PCR. Anal Chem 2015;87:3706-13.
3. O'Sullivan DM, Laver T, Temisak S, et al. Assessing the accuracy of quantitative molecular microbial profiling. Int J Mol Sci 2014;15:21476-91.
